# Radiobiological Effects of Low-Dose Radiation in Normal Fibroblasts of Patients with Head and Neck Cancer Treated with Induction Chemotherapy Combined with Low-Dose Fractionated Radiation

**DOI:** 10.3390/ijms27062525

**Published:** 2026-03-10

**Authors:** Gabriela Winiarska, Tomasz Rutkowski, Adam Gądek, Wojciech Fidyk, Magdalena Głowala-Kosińska, Urszula Kacorzyk, Krzysztof Składowski, Dorota Słonina

**Affiliations:** 1 Center for Translational Research and Molecular Biology of Cancer, Maria Sklodowska-Curie National Research Institute of Oncology Gliwice Branch, Gliwice, Ul. Wybrzeże Armii Krajowej 15, 44-102 Gliwice, Poland; gabriela.winiarska@gliwice.nio.gov.pl; 2 1st Radiation and Clinical Oncology Department, Maria Sklodowska-Curie National Research Institute of Oncology, Gliwice Branch, Ul. Wybrzeże Armii Krajowej 15, 44-102 Gliwice, Poland; tomasz.rutkowski@gliwice.nio.gov.pl (T.R.); urszula.kacorzyk@gliwice.nio.gov.pl (U.K.); krzysztof.skladowski@gliwice.nio.gov.pl (K.S.); 3 Radiotherapy Planning Department, Maria Sklodowska-Curie National Research Institute of Oncology, Gliwice Branch, Ul. Wybrzeże Armii Krajowej 15, 44-102 Gliwice, Poland; adam.gadek@gliwice.nio.gov.pl; 4 Department of Bone Marrow Transplantation and Oncohematology, Maria Sklodowska-Curie National Research Institute of Oncology, Gliwice Branch, Ul. Wybrzeże Armii Krajowej 15, 44-102 Gliwice, Poland; wojciech.fidyk@gliwice.nio.gov.pl (W.F.); magdalena.glowala-kosinska@gliwice.nio.gov.pl (M.G.-K.)

**Keywords:** low-dose fractionated radiation, HRS, normal fibroblasts, HNSCC patients, carboplatin, paclitaxel

## Abstract

The aim of the study was to define radiobiological effects of single and fractionated low doses in normal fibroblasts in 40 patients with squamous cell carcinoma of the head and neck (HNSCC) treated with induction chemotherapy combined with low-dose fractionated radiation (LDFR) and to answer the question regarding the role of low-dose hyper-radiosensitivity (HRS) in these effects. HRS status was determined using flow cytometry-based clonogenic survival assay (cells were irradiated with doses 0.1–4 Gy of 6 MV X-rays). Radiobiological effects (cell kill, kinetics of DSB recognition and repair, chemopotentiation) of LDFR 4x0.5 Gy and a single dose of 2, 0.5 and 0.2 Gy were estimated by clonogenic, pATM and γH2AX foci assays. HRS response was demonstrated for normal fibroblasts in 6 of the 40 HNSCC patients. For all assessed biological parameters, significant interindividual differences were observed. The presence of HRS had no effect on the chemopotentiating effects of LDFR 4x0.5 Gy, which were similar to that after 2 Gy. There was also no association between HRS and the maximum number of pATM and γH2AX foci induced by single (0.2, 0.5, 2 Gy) or fractionated low doses 4x0.5 Gy. Significantly higher percentages of residual pATM and γH2AX foci observed after LDFR 4x0.5 Gy than after 2 Gy were independent of HRS. HRS is a rare finding (15%) in normal fibroblasts from HNSCC patients; therefore, it is of rather little importance in healthy late-reacting connective tissues. Moreover, the fibroblast response to single and fractionated low doses (alone or in combination with carboplatin and paclitaxel) appeared more dependent on individual radiosensitivity than on HRS.

## 1. Introduction

Induction chemotherapy (iCH), although it is not a standard approach, is often used in clinical practice for patients with advanced head and neck cancer (HNSCC). Tumor regression is a potential goal of induction treatment to reduce target areas in the second stage of treatment—radiochemotherapy or radiotherapy. However, iCH, especially the most common one, which is based on the combination of docetaxel, cisplatin, and fluorouracil (TPF), carries a risk of severe acute toxicity. Arnold et al. [1,2] were the first to propose (for locally advanced HNSCC) a new induction treatment in which a three-drug TPF regimen was replaced by two drugs in combination with low-dose fractionated radiation (LDFR). Since then, three further clinical trials have been conducted [3,4,5], including one at our institute [5], in patients with locally advanced HNSCC. The benefit of combining LDFR with drugs such as taxanes, carboplatin, anthracycline, temozolomide, or FOLFIRI-bevacizumab has also been studied in patients with breast, lung, cervical, and brain tumors (for review see Scirocco et al.) [6]. All these studies reported improved therapeutic outcomes and acceptable toxicity.

It is generally believed that the effectiveness of such treatment is due to the phenomenon of low-dose hyper-radiosensitivity (HRS), in which cells die from excessive sensitivity to doses ≤0.5 Gy [7,8]. It has been shown (in vitro and in vivo) that low-dose fractions (<1 Gy) sensitize HRS-positive (HRS+) cancer cells to cytotoxic drugs more effectively than conventional dose fractions (2 Gy) [9,10,11]. However, such chemopotentiating effects of LDFR were also seen in cancer cells that do not present HRS, which undermines its role as the main factor [10,12]. On the contrary, it is not known whether the enhancing effects of LDFR also apply to normal cells and depend on the presence of the HRS effect and whether this translates into a clinical response in normal tissues. The fact that our institute has started a phase II clinical trial [5] using iCH (carboplatin and paclitaxel) combined with LDFR (0.5 Gy fractions) in patients with locally advanced HNSCC gave us a unique opportunity to study the radiobiological effects of LDFR, including chemopotentiation, in normal fibroblasts derived from these patients and to answer the question regarding the role of the HRS phenomenon in these effects.

According to the theory named RIANS (radiation-induced ATM nucleoshuttling), the recognition and repair of radiation-induced DNA double-strand breaks (DSBs) depends on pATM (phosphorylated ataxia telangiectasia mutated) protein that diffuses from the cytoplasm to the nucleus and phosphorylates histone H2AX at the DSB site [13,14]. The authors of RIANS theory, using fluorescence pATM foci assay, found a significant correlation between the maximum number of pATM foci visible after a dose of 2 Gy and side effects in cancer patients [15,16]. According to the RIANS model, low radiation doses (≤0.5 Gy) are too small to produce enough pATM forms to shuttle to the nucleus and recognize DSBs. As a result, some DSBs are not recognized and become lethal [14]. Because the role of RIANS in the effects of low doses including HRS has never been studied before, we also report here the kinetics of recognition and repair of DSBs (estimated by pATM and γH2AX foci) induced by single (0.2 and 0.5 Gy) and fractionated low doses (4x0.5 Gy).

In this study, we present for the first time comprehensive radiobiological data on the effects (cell kill, DSB recognition and repair, chemopotentiation) of low-dose radiation, including the role of the HRS phenomenon, in normal fibroblasts from 40 patients with advanced HNSCC.

## 2. Results

### 2.1. HRS Is a Rare Effect in Normal Fibroblasts of Patients with HNSCC

The raw survival data obtained in the dose–response study for fibroblasts in 40 HNSCC patients are summarized in Table 1. The radiosensitivity of fibroblasts at low and high doses (from 0.1 Gy to 4 Gy) varied significantly between cancer patients (*p* < 0.00001). For example, the mean surviving fraction at 0.2 Gy (SF0.2) ranged from 0.59 to 1.00, at 0.5 Gy (SF0.5) from 0.57 to 0.88, and at 2 Gy (SF2) from 0.17% to 0.44.

**Figure 1:**
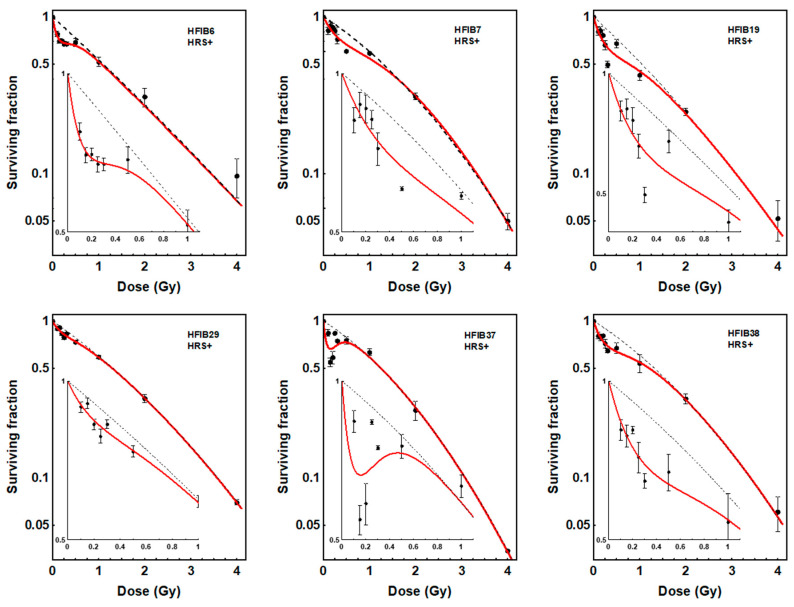
Dose–response curves for survival of fibroblasts (HFIB) from 6 patients showing low-dose hyper-radiosensitivity (HRS+). Each point represents the mean ± SEM of 2–3 experiments. Red solid lines show the fits to the data of the induced-repair (IR) model. Black dotted lines show the fits to the data of the linear quadratic model (LQ). The insets show the low-dose region and demonstrate the increased effectiveness of doses <0.5 Gy compared to the LQ model prediction.

To recognize low-dose hyper-radiosensitivity, the induced-repair (IR) model [17,18] was fitted to the survival data of each patient and the dose–response curves were compared with the linear quadratic (LQ) model prediction. As a result, the presence of the HRS effect (increased cell kill at low doses compared to LQ model prediction) was found for fibroblasts in 6 of the 40 patients (Figure 1). The values of parameters of the IR and LQ models for the six HRS-positive patients are shown in Table 2. For fibroblasts of the remaining 34 patients, the dose–response relationship was linear quadratic without deviation. Interestingly, HRS-positive cells were significantly more radioresistant to a dose of 2 Gy than HRS-negative fibroblasts (mean SF2 = 0.29 vs. 0.25, respectively, *p* < 0.0001).

### 2.2. Similar Levels of Cell Death Induced by LDFR 4x0.5 Gy and a Single Dose of 2 Gy, Independent of HRS

**Figure 2:**
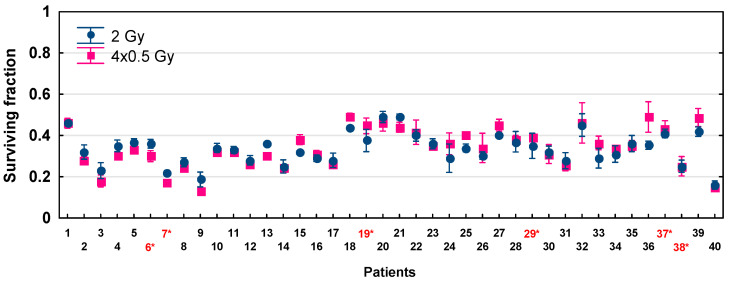
Fibroblast survival after exposure to LDFR 4x0.5 Gy versus a single dose of 2 Gy for 40 HNSCC patients. Each point represents the mean ± SEM of 2–3 experiments. Red numbers with an asterisk indicate patients with HRS-positive fibroblasts.

The biological effects (cell survival) of LDFR 4x0.5 Gy compared to a single dose of 2 Gy measured in fibroblasts in 40 HNSCC patients are presented in Figure 2 and Table 3. For each patient, cell death induced by LDFR 4x0.5 Gy was not significantly different from that induced by a single dose of 2 Gy (*p* = 0.8561). The mean SF after 2 Gy ranged from 0.19 to 0.49 while after LDFR 4x0.5 Gy from 0.13 to 0.49. Therefore, no inverse effect of fractionation was observed even in HRS-positive fibroblasts (*p* = 0.9492).

**Figure 3:**
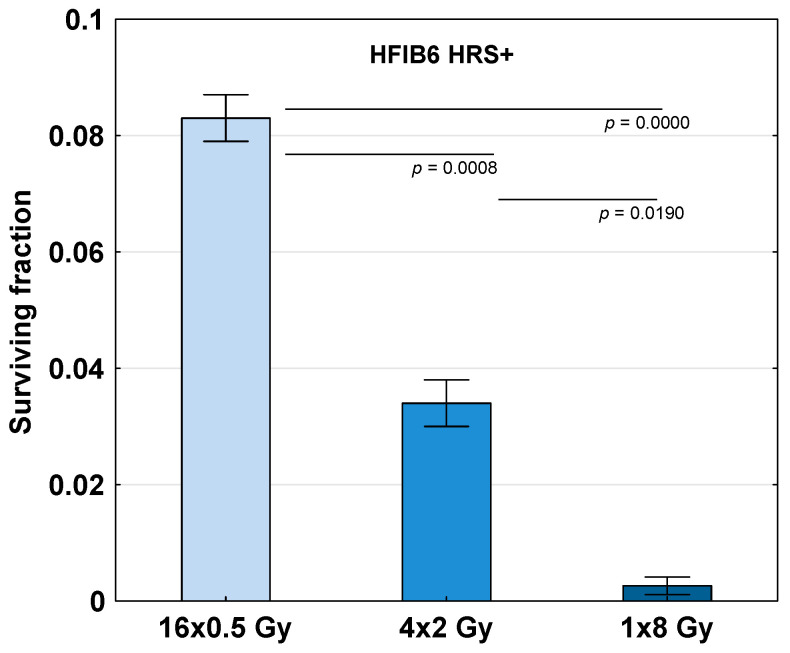
Survival of HRS-positive fibroblasts in one patient (HFIB6) exposed to a total dose of 8 Gy given as 16 fractions of 0.5 Gy (two fractions a day), 4 fractions of 2 Gy (one fraction a day) and a single dose of 8 Gy. Bars represent the mean ± SEM of 2 experiments.

To further recognize the role of HRS in the LDFR regimen used in our clinical trial, the HRS-positive fibroblasts in one patient (HFIB6) were subjected to a total dose of 8 Gy given as LDFR 16x0.5 Gy (two fractions a day) compared to 4x2 Gy (once a day) and a single dose of 8 Gy. The data shown in Figure 3 demonstrate a significant sparing effect of LDFR 16x0.5 Gy, (SF = 0.08 ± 0.004) compared to two other regimens 4x2 Gy (SF = 0.03 ± 0.004, *p* < 0.001) and 1x8 Gy (SF = 0.003 ± 0.001, *p* < 0.00001).

### 2.3. Similar Chemopotentiating Effects of LDFR 4x0.5 Gy and 2 Gy on Carboplatin and Paclitaxel, Independent of HRS

**Figure 4:**
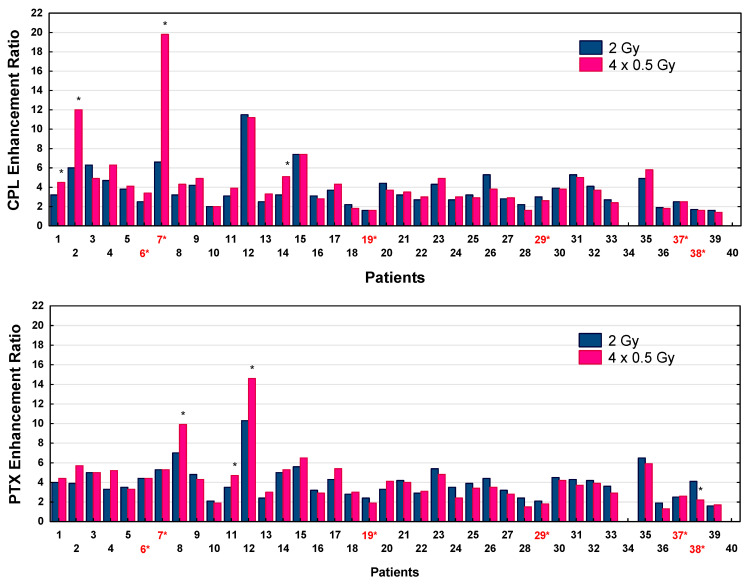
Carboplatin (CPL) and paclitaxel (PTX) enhancement ratios (SF) by LDFR 4x0.5 Gy versus a single dose of 2 Gy for 38 patients. * *p* < 0.05. Red numbers with an asterisk indicate patients with HRS-positive fibroblasts.

The effects of LDFR 4x0.5 Gy versus 2 Gy on carboplatin and paclitaxel quantified according to the surviving fractions are summarized in Table 3. In the case of all 38 studied patients, LDFR 4x0.5 Gy as well as a single dose of 2 Gy enhanced cytotoxic effects of carboplatin and paclitaxel were seen as a significant (*p* < 0.0001) reduction in cell survival compared to the drug alone. However, in all but seven patients, the chemopotentiating effects induced by the two radiation regimens were not significantly different. The carboplatin and paclitaxel enhancement ratios (ERs) are presented in Figure 4. Significantly higher carboplatin ERs after LDFR 4x0.5 Gy than after a single dose of 2 Gy were obtained in fibroblasts in four patients (*p* < 0.05), only one of whom was HRS-positive (HFIB7). For him, the carboplatin ER reached its maximum after LDFR 4x0.5 Gy (19.8 vs. 6.6 for 2 Gy, *p* < 0.05). Potentiating effects of LDFR 4x0.5 Gy on paclitaxel were significantly higher than those after 2 Gy in fibroblasts in three other patients (*p* < 0.05) but none of them were HRS-positive.

### 2.4. Less pATM and γH2AX Foci Were Induced by LDFR 4x0.5 Gy than by a Single Dose of 2 Gy

To test the role of DSB recognition in the biological effects of LDFR 4x0.5 Gy versus a single dose of 2 Gy, and after a single low dose of 0.2 and 0.5 Gy, we analyzed induction of pATM and γH2AX foci in fibroblasts in 36 HNSCC patients. First, for each patient and dose the kinetics of DSB appearance and disappearance was assessed to find the individual time point with the maximum number of pATM and γH2AX foci (Figure S1). The time to DSB recognition was similar for all patients and for most of them the maximum number of pATM and γH2AX foci was visible at 30 min after irradiation.

**Figure 5:**
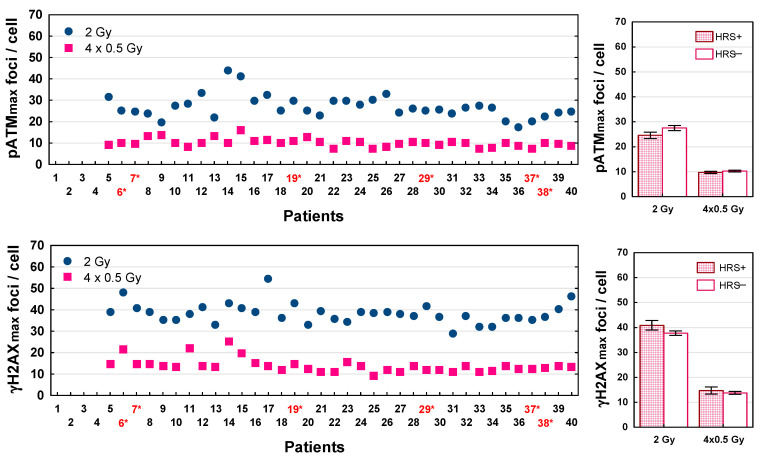
Maximum number of pATM (pATMmax) and γH2AX (γH2AXmax) foci induced by LDFR 4x0.5 Gy versus a single dose of 2 Gy in fibroblasts in 36 patients (**left graphs**). Red numbers with an asterisk indicate patients with HRS-positive fibroblasts. (**Right graphs**) depict comparison between HRS-positive (HRS+) fibroblasts in 6 patients and HRS-negative (HRS−) fibroblasts in 30 patients.

**Figure 6:**
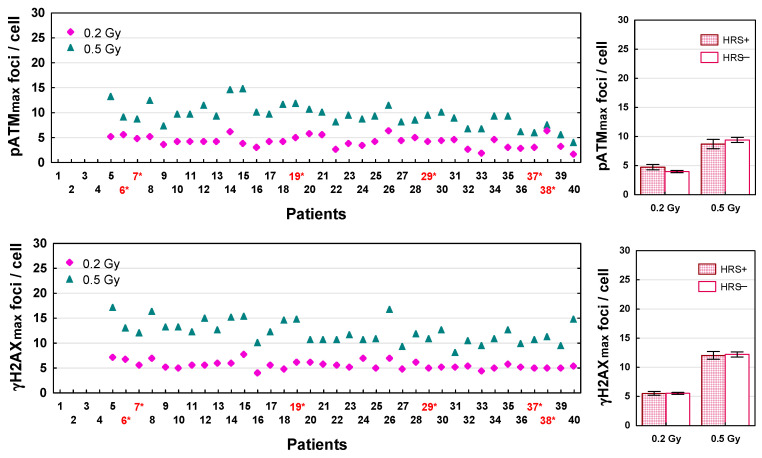
Maximum number of pATM and γH2AX foci induced by a single dose of 0.2 Gy and 0.5 Gy in fibroblasts in 36 patients (**left graphs**). Red numbers with an asterisk indicate patients with HRS-positive fibroblasts. (**Right graphs**) depict comparison between HRS-positive (HRS+) fibroblasts in 6 patients and HRS-negative (HRS−) fibroblasts in 30 patients.

The maximum numbers of pATM and γH2AX foci recognized after LDFR 4x0.5 Gy versus a single dose of 2 Gy for each patient are presented in Figure 5. The numbers of radiation induced DSBs (both types of foci) differed significantly between 36 patients (*p* < 0.0001). Interestingly, the maximum numbers of pATM and γH2AX foci recognized after LDFR 4x0.5 Gy were 2–4 times lower (ranged from 7 to 16 and 9 to 25 foci per cell, respectively) than those after a single dose of 2 Gy (that ranged from 17 to 44 and from 29 to 54, respectively) (*p* < 0.0001) but very similar to those after a single dose of 0.5 Gy (Figure 6). This relationship was seen in fibroblasts of all patients irrespective of HRS status. Both groups of fibroblasts (HRS-positive in 6 patients and HRS-negative in 30 patients) showed on average similar maximum numbers of pATM and γH2AX foci per cell (*p* > 0.05) after a single dose of 2 Gy (25 vs. 27, and 41 vs. 38) and after LDFR 4x0.5 Gy (10 vs. 10 and 15 vs. 14) respectively (Figure 5). Such a lack of difference in the maximum number of both types of foci between HRS-positive and HRS-negative fibroblasts (*p* > 0.05) was also seen after a single low dose of 0.2 Gy (4 vs. 5 and 6 vs. 6) and 0.5 Gy (9 vs. 9 and 12 vs. 12) respectively (Figure 6). 

### 2.5. More Residual pATM and γH2AX Foci After LDFR 4x0.5 Gy than After a Single Dose of 2 Gy

**Figure 7:**
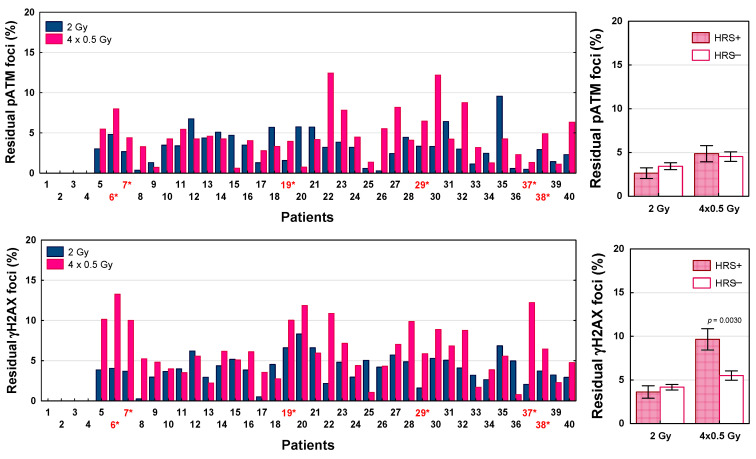
The percentage of residual pATM and γH2AX foci scored 24 h after LDFR 4x0.5 Gy versus a single dose of 2 Gy in fibroblasts in 36 patients (**left graphs**). Red numbers with an asterisk indicate patients with HRS-positive fibroblasts. (**Right graphs**) depict comparison between HRS-positive (HRS+) fibroblasts in 6 patients and HRS-negative (HRS−) fibroblasts in 30 patients.

**Figure 8:**
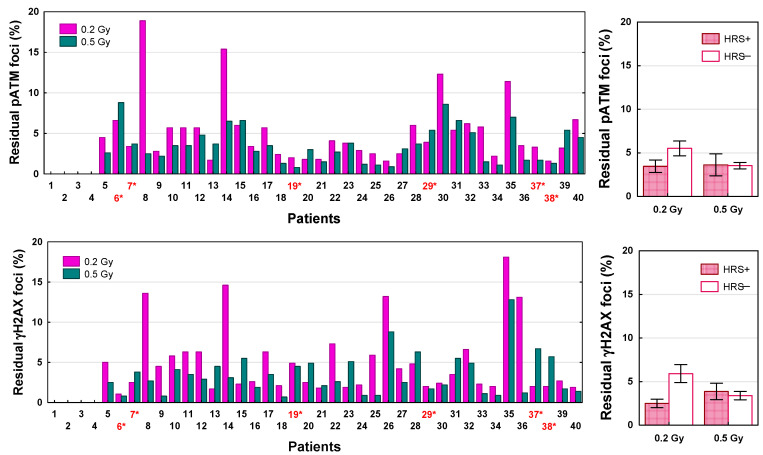
The percentage of residual pATM and γH2AX foci scored 24 h after a single dose of 0.2 Gy and 0.5 Gy in fibroblasts in 36 patients (**left graphs**). Red numbers with an asterisk indicate patients with HRS-positive fibroblasts. (**Right graphs**) depict comparison between HRS-positive (HRS+) fibroblasts in 6 patients and HRS-negative (HRS−) fibroblasts in 30 patients.

To test the role of DSB repair in the biological effects of LDFR 4x0.5 Gy versus a single dose of 2 Gy and after a single low dose of 0.2 and 0.5 Gy, we analyzed the number of residual pATM and γH2AX foci scored 24 h after irradiation. DSB repair efficiency was expressed as the percentage of residual foci relative to the maximum number of radiation-induced foci. Results for 36 patients are shown in Figure 7 and Figure 8. For each radiation dose, the percentage of residual pATM and γH2AX foci differed significantly between 36 patients (*p* < 0.0001). In the case of pATM foci, the percentage of unrepaired DSBs after LDFR 4x0.5 Gy ranged from 0.6% to 12.5% and for 23 patients including six who were HRS- positive it was significantly higher (mean 5.4%) than after 2 Gy (mean 2.5%, *p* < 0.0001). Similarly, the percentage of residual γH2AX foci after LDFR 4x0.5 Gy (0.8–13.3%) for 25 patients including six who were HRS-positive was significantly higher (mean 7.5%) than for 2 Gy (mean 3.8%, *p* < 0.0001). Comparison of the mean percentage of unrepaired DSBs between HRS-positive and HRS-negative fibroblasts showed significant difference only in the case of γH2AX foci after LDFR 4x0.5 Gy. HRS-positive fibroblasts presented a significantly (*p* < 0.005) higher percentage of γH2AX residual foci (9.6%) than HRS-negative cells (5.5%) (Figure 7). Surprisingly, differences in the percentage of residual pATM and γH2AX foci between HRS-positive and HRS-negative fibroblasts were not significant after a single low dose of 0.2 and 0.5 Gy (Figure 8).

## 3. Discussion

In this study, we present for the first time comprehensive radiobiological data on the effects (cell kill, DSB recognition and repair, chemopotentiation) of single (0.2 and 0.5 Gy) and fractionated low doses (4x0.5 Gy), including the role of the HRS phenomenon, in normal fibroblasts from 40 patients with advanced HNSCC. These patients are enrolled in a phase II clinical trial aimed at assessing the efficacy and toxicity of induction chemotherapy combined with LDFR at 0.5 Gy. For all assessed biological parameters, we observed statistically significant interindividual differences, however independent of HRS. These results therefore provide a basis for future investigation of the relationship between these radiobiological parameters (biomarkers) and the clinical radiosensitivity of healthy tissues in these patients.

The present data shows that HRS is a rare effect in normal fibroblasts of patients with HNSCC. Out of 40 patients, only fibroblasts in six patients (15%) showed a HRS effect (Figure 1, Table 2). These findings are consistent with a previous study [20] demonstrating HRS response in fibroblasts in 4 of 25 (16%) patients with cervix cancer. On the contrary, studies on human tumor cell lines have shown that 75% of them are HRS-positive [21], especially those (although not all) that are known to be resistant to conventional radiotherapy doses (2 Gy) [19,22,23]. Therefore, human fibroblasts, being sensitive to high doses of radiation (SF2 < 0.5), may be less prone to low doses. In the present study, HRS-positive fibroblasts were significantly more radioresistant to a dose of 2 Gy than HRS-negative fibroblasts (SF2 = 0.29 vs. 0.25), which confirms the relationship between SF2 and HRS. If these in vitro data translate to cancer patients, the fact that HRS is much more common in cancer cells than in relevant healthy tissues could potentially improve the therapeutic ratio.

In the present study, both HRS-positive and HRS-negative fibroblasts in 40 HNSCC patients showed a similar response (SF) to LDFR 4x0.5 Gy and a single dose of 2 Gy (Figure 2). In the case of HRS-negative fibroblasts (in 34 patients) the expected sparing effect of fractionation was not observed. On the other hand, HRS-positive fibroblasts, even those with the strongest HRS (HFIB37 with α_s_/α_r_ = 18.4), did not demonstrate the expected hyper-radiosensitivity after LDFR 4x0.5 Gy. In fact, HRS-positive fibroblasts (HFIB6) showed a significant sparing effect of fractionation after LDFR 16x0.5 Gy compared to 4x2 Gy and a single dose of 8 Gy. This fact suggests that prolonged overall irradiation time (8 days) stopped the HRS response. To our knowledge, there are no other cell survival data on the effect of multiple low doses in normal human fibroblasts. The only other studies are cytogenetic studies [24] that showed HRS-positive fibroblasts demonstrate the sparing effect of fractionation (lower micronucleus induction) after LDFR 3x0.5 Gy versus a single dose of 1.5 Gy. Thus, the presence of HRS in normal fibroblasts (target cells responsible for radiation-induced fibrosis) is of rather little importance in late-reacting connective tissues.

The data concerning the role of HRS in human tumor cells are not homogenous. Short et al. [25] reported significantly lower survival of HRS-positive glioma cells (T98G) after LDFR 3x0.4 Gy (at 4h intervals) than after a single dose of 1.2 Gy, and that the inverse effect of fractionation did not disappear even after 15 fractions of 0.4 Gy (given over 5 days) compared to five fractions of 1.2 Gy. Similarly, Gupta et al. [10] observed lower survival in one HRS-positive lung cancer cell line (H-157) after 12 fractions of 0.5 Gy compared to three fractions of 2 Gy. However, in the second HRS-positive lung cancer cell line (UKY-29), they observed the opposite result, namely higher cell survival after 12x0.5 Gy than after 3x2 Gy.

In the current study, for the first time, the role of HRS on the chemopotentiating effects of LDFR was assessed in normal fibroblasts of patients with HNSCC enrolled in the clinical trial using iCH (carboplatin and paclitaxel) combined with LDFR of 0.5 Gy. Generally, the potentiating effects of LDFR 4x0.5 Gy on carboplatin and paclitaxel, although significant, appeared to be similar to a single dose of 2 Gy for all except seven patients. Surprisingly, cells of only one of the seven patients were HRS-positive and presented the least pronounced HRS (HFIB7 with α_s_/α_r_ = 3.9) (Table 2). Interestingly, patients with fibroblasts showing maximum carboplatin ERs after LDFR 4x0.5 Gy were not the patients showing maximum paclitaxel ERs. Thus, this fact confirms that the chemopotentiating effects of LDFR also depend on the cytostatic type [12].

The role of HRS undermines the fact that the chemopotentiating effects of LDFR were also seen in human cancer cells that do not present HRS. In the study of Gupta et al. [10], a HRS-negative human lung cancer cell line (H460) showed a maximum cisplatin ER with LDFR 4x0.5 Gy (expressed as clonogenic inhibition and apoptosis) compared to a single dose of 2 Gy, whereas the most radioresistant HRS-positive lung cancer cells A549 presented the least cisplatin ER with no differences between LDFR and 2 Gy. This finding is consistent with that in cervix cancer cells SiHa and CaSki, in which LDFR 4x0.5 Gy enhanced the effects of cisplatin (but not paclitaxel) significantly more than a single dose of 2 Gy (seen as decreased survival and higher number of residual γH2AX), although they lack HRS [12]. Thus, all these observations provide evidence that the response to LDFR and the chemopotentiation of carboplatin and paclitaxel by LDFR 4x0.5 Gy in normal and tumor cells are more dependent on individual radiosensitivity and other unknown factors than on HRS.

It is widely accepted that 10–20% of the patients developed toxicity following radiotherapy, and the identification of such patients with reliably predictive assays is needed. It is generally believed that individual radiosensitivity depends on the efficiency of DSB repair and that the less-efficient repair of radiation-induced DSBs may be a critical factor in radiation toxicity [26]. The immunofluorescence pATM and γH2AX foci assays proved to be highly sensitive methods for determining the induction and repair of DSBs induced by low or high radiation doses [27,28,29]. Recent studies show [29,30] significant correlation between surviving fractions and numbers of residual pATM and γH2AX foci (visible 24 h after irradiation) in normal fibroblasts of cancer patients. To understand the lack of difference in fibroblast survival between LDFR 4x0.5 Gy and a single dose of 2 Gy we examined the kinetics of pATM and γH2AX foci’s appearance and disappearance after these doses (and also after a single dose of 0.2 and 0.5 Gy) in fibroblasts in 36 patients. The maximum numbers of pATM and γH2AX foci induced after each of these doses varied significantly between cancer patients (Figure 5 and Figure 6). This result is not consistent with the common belief that the same radiation dose induces the same number of DSBs (about 40 DSBs per Gy) [26,27] but on the other hand it supports the thesis that the visible foci are only those DSBs that were recognized by the pATM phosphorylation of H2AX histone with a chance to be repaired by non-homologous end-joining. According to the RIANS model, the rate of recognized DSBs represents an individual characteristic and is proportional to the number of pATM monomers that shuttle from the cytoplasm to the nucleus [14]. Recently, the maximum number of pATM foci visible after 2 Gy has been shown to be a reliable predictor of clinical radiosensitivity [15,16].

Interestingly, the maximum numbers of pATM and γH2AX foci recognized after LDFR 4x0.5 Gy were similar to those after a single dose of 0.5 Gy. This fact suggests that after each fraction of 0.5 Gy, the most DSBs were repaired in the intervals (at least 6 h) between the four fractions of 0.5 Gy. Indeed, using HRS-positive (HFIB6) and HRS- negative (HFIB16) fibroblasts, we observed the same maximum number of pATM and γH2AX foci after each of the four consecutive 0.5 Gy doses, although cell survival decreased with each subsequent dose (Figure S2). The lack of significant difference in the maximum numbers of pATM and γH2AX foci between HRS-positive and HRS-negative fibroblasts (Figure 5 and Figure 6) undermines the role of RIANS in the HRS effect and is consistent with the previous data showing no HRS response for initial pATM and γH2AX foci [29].

On the contrary, the present analysis of the number of residual pATM and γH2AX foci assessed 24 h after irradiation showed, in most patients, including all with HRS-positive fibroblasts, a significantly higher percentage of unrepaired DSBs after LDFR 4x0.5 than after a single dose of 2 Gy. The results were independent of HRS, although in the case of LDFR 4x0.5 Gy the number of residual γH2AX (but not pATM) foci was significantly higher in HRS-positive compared to HRS-negative fibroblasts. Previously, HRS after low doses was demonstrated for residual γH2AX foci in human fibroblasts [29,31] and in the epidermal skin of radiotherapy patients [32]. Thus, all these observations support the leading role of DSB repair in response to low and high doses of radiation. Recently, Nuijens et al. [33] found the efficiency of DSB repair expressed as the γH2AX foci decay ratio (FDR) in lymphocytes to be a strong predictive factor of late radiation toxicity in patients with prostate cancer.

## 4. Materials and Methods

### 4.1. Primary Cell Cultures

Primary normal fibroblasts (HFIB) from 40 patients with locally advanced squamous cell carcinoma of the head and neck (HNSCC) were used. The patients are enrolled in a clinical trial on the effectiveness and toxicity of induction chemotherapy combined with LDFR [5]. The group consisted of 32 (80%) men and 8 (20%) women with a median age of 60 years. In most cases, primary tumor site was oropharynx (80%), hypopharynx (13%) or larynx (7%). All patients presented advanced stage III or IV. Two cycles of iCH consisting of carboplatin (AUC 6) and paclitaxel (75 mg/m^2^) combined with LDFR 2x0.5 Gy on days 1, 8 and 15 were given. Additionally, two doses of 0.5 Gy were given on day 2. Total dose administered to patients was 8 Gy. After signed informed consent, skin biopsies were taken from the inside of the patient’s forearm before start of treatment. The current study was approved by Ethics Committee of Maria Sklodowska-Curie National Research Institute of Oncology, Gliwice branch (decision code: KB/430-106/19, date of approval: 19 November 2019).

The procedure for obtaining and preparing the cells was described previously [24,34]. The cells were grown as a monolayer in 75-cm^2^ culture flasks (Falcon, Durham, NC, USA) containing Dulbecco’s Modified Eagle Medium (DMEM, Biowest, Nuaille, France) supplemented with 10% fetal bovine serum (FBS; Gibco, Grand Island, NY, USA), 1% HEPES, 1% sodium pyruvate (Biowest, Nuaille, France) and antibiotics (100 U/mL penicillin, 100 μg/mL streptomycin, and 0.25 μg/mL amphotericin B; Biowest, Nuaille, France). All cells were incubated at 37 °C and 5% CO_2_.

### 4.2. Cell Treatments

For cell treatments, carboplatin (10 mg/mL; Carbomedac, Wedel, Germany) and paclitaxel (6 mg/mL; Accord Healthcare, Warsaw, Poland) were used in concentration that inhibits fibroblast survival by ~50% (IC50)—0.4 μg/mL and 0.5 nM, respectively. Irradiation was performed using a 6-MV X-ray beam generated by TrueBeam linear accelerator (Varian). For irradiation, 100 mm Petri dishes with cells were put into the poly(methyl-methacrylate) block (30 × 30 cm). The virtual dose distribution was calculated using the Acuros v. 16.1.0 algorithm in the Eclipse treatment planning system (Varian, Palo Alto, CA, USA). The homogeneity of dose was ±1.5%. The dose rate was 300 MU/minute (1 cGy/MU).

Flow cytometry-based clonogenic survival assay was performed to investigate individual radiosensitivity (on doses ranging from 0.1 to 4 Gy) and HRS status.

Standard clonogenic assay was utilized to compare surviving fractions (SFs) of LDFR 4x0.5 Gy versus a single dose of 2 Gy, and to evaluate chemopotentiating effects of these two modes of irradiation on carboplatin and paclitaxel. For combined treatments, experiments were performed as follows: 24 h after plating, the cells were treated with carboplatin (0.4 μg/mL) or paclitaxel (0.5 nM) and 24 h later (without changing the medium) they were irradiated with a single dose of 2 Gy (one set of cells) and 4 fractions of 0.5 Gy (second set of cells), given during two days with at least 6 h interval between two fractions per day. In a separate experiment, HRS-positive fibroblasts in one patient were exposed to a total dose of 8 Gy given as 16 fractions of 0.5 Gy (two fractions a day with 6 h interval), 4 fractions of 2 Gy (one fraction a day) and a single dose of 8 Gy.

pATM and γH2AX foci assays were used to compare kinetics of DSB recognition and repair after irradiation with single doses of 0.2, 0.5 and 2 Gy and LDFR 4x0.5 Gy.

### 4.3. Standard and Flow Cytometry-Based Clonogenic Survival Assays

For the standard clonogenic survival assay, exponentially growing cells were seeded onto 100 mm Petri dishes (1000–2000 cells per dish) with 15 mL of culture medium. For the flow cytometry-based clonogenic survival assay, the cells were resuspended in a single cell suspension in 3 mL of DMEM with 10% FBS (at a density of ~1 × 10^6^/mL). Next, cells were sorted using a cytometer FACSAriaTM III Cell Sorter (Becton Dickinson Science, San Diego, CA, USA) and the exact number of cells (2000) was dispensed directly onto 100 mm Petri dishes with 15 mL of culture medium. Three Petri dishes were prepared for each dose. The cells were irradiated the next day. All sorts and irradiations were carried out at room temperature (22 ± 2 °C). After incubation at 37 °C for 12–14 days, the resultant colonies were stained with 1% crystal violet (Merck, Darmstadt, Germany) and those with more than 50 cells were scored. The surviving fraction (SF) at each dose point (an average of three plates) was calculated as the ratio of the plating efficiencies for irradiated and unirradiated cells.

The carboplatin or paclitaxel enhancement ratio (ER) by radiation was calculated using the formula: ER = SF of cytostatic drug alone/SF of cytostatic drug + radiation.

### 4.4. pATM and γH2AX Foci Assays

Fibroblasts were seeded onto coverslips placed in 100 mm Petri dishes (one coverslip for each treatment point) and irradiated when confluent. After irradiation (10, 20, 30, 60 min and 24 h), cells were fixed in 4% paraformaldehyde (AppliChem, Darmstadt, Germany) for 15 min, permeabilized in 0.5% Triton X-100 (Sigma-Aldrich, Shnelldorf, Germany) in buffer solution pH 6.8 (Chempur, Piekary Śląskie, Poland) for 10 min, and blocked in 4% bovine serum albumin (Carl Roth, Karlsruhe, Germany) in PBS (Biowest, Nuaille, France) for 30 min at 37 °C. Then cells were incubated with primary antibody against pATM or γH2AX for 1 h at room temperature. Anti-ATM Protein Kinase pS1981 antibody (Rockland, Limerick, PA, USA) was applied at 1:500, and Anti-γ-H2AX (phospho S139) antibody [9F3] (Abcam, Cambridge, UK) at 1:1000. Next, cells were incubated with Alexa Fluor 488-conjugated goat anti-mouse IgG H&L secondary antibody (1:500; Abcam, Cambridge, UK) for 1 h at room temperature. Cells were mounted and counterstained with Vectashield Antifade Mounting Medium with DAPI (Vector Laboratories, Newark, CA, USA). Fluorescence images were analyzed using fluorescence microscopy AxioImager M2 and the super-resolution system ELYRA 7 with Lattice SIM (Carl Zeiss, Oberkochen, Germany). For each patient, foci were counted in 100–200 cell nuclei per slide. For all analyses, spontaneous (0 Gy) pATM or γH2AX foci were subtracted from those after irradiation.

### 4.5. Statistical Analysis

The clonogenic survival data for doses ranging from 0.1 Gy to 4 Gy for each patient were fitted to the linear quadratic (LQ) model:

SF = exp·(− α_d_− β_d_^2^) (1)

and to the induced-repair (IR) model [17,18]:

SF = exp·[−α_r_(1 + (α_s_/α_r_−1)·e^−d/dc^)·d − β_d_^2^] (2)

where d is dose, α_r_ is α extrapolated from the high-dose response, and α_s_ is α derived from the response at low doses; α_s_ > α_r_ (the confidence limits of which do not overlap) represents increased sensitivity at low doses, and dc is the dose of transition from HRS to IRR (induced radioresistance) [19]. The data were fitted using nonlinear least-squares regression applying the iterative method of Gauss–Newton (Statistica 13.3). Analysis of variance and unpaired Student’s t test were used to compare means. A *p* value < 0.05 was considered statistically significant.

## 5. Conclusions

The present data provide evidence that HRS is a rare finding (15%) in normal fibroblasts from HNSCC patients, therefore it is of rather little importance in healthy late-reacting connective tissues. Moreover, the fibroblast response to single and fractionated low doses (alone or in combination with carboplatin and paclitaxel) appeared more dependent on individual radiosensitivity (that depends on the efficiency of DSB recognition by pATM and repair) than on HRS. This warrants an evaluation of the predictive value of these radiobiological parameters for radiation toxicity in the HNSCC patients.

## Figures and Tables

**Table 1:** Cell survival (SF) after irradiation with single doses ranging from 0.1 to 4 Gy for fibroblasts in 40 patients with head and neck cancer. Each value is mean ± SEM. Patients with numbers preceded by “H” show low-dose hyper-radiosensitivity

Patients No.	0.1 Gy	0.15 Gy	0.2 Gy	0.25 Gy	0.3 Gy	0.5 Gy	1 Gy	2 Gy	4 Gy
1.	0.86 ± 0.01	0.96 ± 0.03	0.99 ± 0.01	1.00 ± 0.00	1.00 ± 0.00	0.88 ± 0.02	0.45 ± 0.05	0.24 ± 0.01	0.04 ± 0.004
2.	0.89 ± 0.03	0.84 ± 0.05	0.73 ± 0.07	0.75 ± 0.06	0.80 ± 0.06	0.65 ± 0.06	0.41 ± 0.03	0.17 ± 0.02	0.04 ± 0.002
3.	0.90 ± 0.04	0.93 ± 0.03	0.84 ± 0.04	0.80 ± 0.03	0.75 ± 0.05	0.60 ± 0.04	0.38 ± 0.03	0.22 ± 0.01	0.02 ± 0.004
4.	0.88 ± 0.04	0.93 ± 0.02	0.91 ± 0.02	0.91 ± 0.02	0.84 ± 0.01	0.75 ± 0.03	0.56 ± 0.05	0.34 ± 0.02	0.07 ± 0.010
5.	0.95 ± 0.02	0.88 ± 0.02	0.81 ± 0.04	0.85 ± 0.05	0.83 ± 0.04	0.74 ± 0.02	0.53 ± 0.02	0.21 ± 0.01	0.04 ± 0.009
H6.	0.77 ± 0.03	0.70 ± 0.02	0.70 ± 0.02	0.67 ± 0.02	0.67 ± 0.02	0.69 ± 0.04	0.52 ± 0.04	0.31 ± 0.04	0.10 ± 0.027
H7.	0.82 ± 0.05	0.87 ± 0.05	0.86 ± 0.05	0.82 ± 0.03	0.72 ± 0.05	0.60 ± 0.01	0.59 ± 0.01	0.31 ± 0.02	0.05 ± 0.006
8.	0.93 ± 0.03	0.86 ± 0.02	0.79 ± 0.03	0.86 ± 0.02	0.81 ± 0.03	0.63 ± 0.01	0.51 ± 0.02	0.20 ± 0.01	0.03 ± 0.005
9.	0.96 ± 0.04	0.87 ± 0.07	0.86 ± 0.03	0.75 ± 0.04	0.83 ± 0.04	0.77 ± 0.06	0.54 ± 0.03	0.18 ± 0.01	0.03 ± 0.017
10	0.81 ± 0.04	0.94 ± 0.04	0.98 ± 0.02	0.82 ± 0.07	0.91 ± 0.02	0.79 ± 0.06	0.60 ± 0.06	0.30 ± 0.04	0.05 ± 0.010
11	0.96 ± 0.03	0.99 ± 0.01	0.85 ± 0.06	0.86 ± 0.06	0.94 ± 0.03	0.80 ± 0.04	0.54 ± 0.07	0.26 ± 0.03	0.05 ± 0.009
12.	0.90 ± 0.03	0.84 ± 0.03	0.83 ± 0.02	0.76 ± 0.04	0.80 ± 0.02	0.70 ± 0.05	0.50 ± 0.03	0.18 ± 0.02	0.03 ± 0.002
13.	0.95 ± 0.01	0.91 ± 0.01	0.91 ± 0.01	0.84 ± 0.02	0.90 ± 0.03	0.83 ± 0.06	0.61 ± 0.02	0.25 ± 0.03	0.06 ± 0.000
14.	0.90 ± 0.02	0.91 ± 0.02	0.87 ± 0.02	0.85 ± 0.02	0.87 ± 0.02	0.83 ± 0.02	0.67 ± 0.02	0.34 ± 0.03	0.09 ± 0.017
15.	0.83 ± 0.04	0.78 ± 0.09	0.74 ± 0.05	0.79 ± 0.03	0.72 ± 0.05	0.71 ± 0.05	0.41± 0.03	0.17 ± 0.01	0.04 ± 0.007
16.	0.99 ± 0.01	0.94 ± 0.03	0.98 ± 0.02	0.95 ± 0.02	0.76 ± 0.07	0.77 ± 0.03	0.52 ± 0.03	0.27 ± 0.04	0.04 ± 0.012
17.	0.86 ± 0.04	0.89 ± 0.03	0.90 ± 0.05	0.93 ± 0.02	0.82 ± 0.05	0.66 ± 0.09	0.45 ± 0.08	0.18 ± 0.03	0.05 ± 0.011
18.	0.95 ± 0.02	0.93 ± 0.03	0.89 ± 0.04	0.85 ± 0.04	0.88 ± 0.05	0.67 ± 0.02	0.52 ± 0.04	0.27 ± 0.04	0.09 ± 0.027
H19.	0.81 ± 0.05	0.82 ± 0.05	0.76 ± 0.06	0.66 ± 0.04	0.50 ± 0.02	0.68 ± 0.05	0.42 ± 0.03	0.25 ± 0.01	0.05 ± 0.015
20.	0.97 ± 0.01	0.84 ± 0.06	0.92 ± 0.05	0.89 ± 0.03	0.88 ± 0.02	0.75 ± 0.03	0.56 ± 0.01	0.22 ± 0.01	0.07 ± 0.013
21.	0.87 ± 0.02	0.81 ± 0.03	0.84 ± 0.01	0.86 ± 0.01	0.75 ± 0.04	0.80 ± 0.02	0.56 ± 0.04	0.27 ± 0.03	0.06 ± 0.009
22.	0.84 ± 0.08	0.85 ± 0.01	0.75 ± 0.04	0.85 ± 0.10	0.93 ± 0.01	0.73 ± 0.08	0.61 ± 0.01	0.21 ± 0.01	0.05 ± 0.000
23.	0.86 ± 0.01	0.80 ± 0.02	0.91 ± 0.01	0.86 ± 0.03	0.82 ± 0.01	0.79 ± 0.01	0.57 ± 0.01	0.17 ± 0.02	0.02 ± 0.002
24.	0.92 ± 0.04	0.83 ± 0.03	0.83 ± 0.04	0.85 ± 0.03	0.82 ± 0.03	0.57 ± 0.06	0.42 ± 0.05	0.19 ± 0.02	0.03 ± 0.008
25.	0.97 ± 0.02	0.97 ± 0.03	0.96 ± 0.04	0.91 ± 0.05	0.88 ± 0.02	0.85 ± 0.02	0.53 ± 0.03	0.23 ± 0.03	0.04 ± 0.013
26.	0.93 ± 0.05	0.88 ± 0.01	0.96 ± 0.02	0.85 ± 0.05	0.88 ± 0.02	0.83 ± 0.04	0.58 ± 0.02	0.30 ± 0.02	0.05 ± 0.005
27.	0.94 ± 0.01	0.87 ± 0.06	0.86 ± 0.03	0.88 ± 0.03	0.82 ± 0.03	0.76 ± 0.03	0.56 ± 0.03	0.29 ± 0.02	0.08 ± 0.007
28.	0.79 ± 0.06	0.66 ± 0.05	0.69 ± 0.04	0.70 ± 0.05	1.00 ± 0.00	0.81 ± 0.04	0.61 ± 0.01	0.27 ± 0.05	0.04 ± 0.015
H29.	0.89 ± 0.02	0.91 ± 0.02	0.83 ± 0.02	0.79 ± 0.02	0.83 ± 0.02	0.74 ± 0.02	0.59 ± 0.02	0.32 ± 0.02	0.07 ± 0.003
30.	0.98 ± 0.01	0.95 ± 0.03	0.91 ± 0.01	0.71 ± 0.04	0.69 ± 0.01	0.59 ± 0.04	0.63 ± 0.03	0.27 ± 0.01	0.03 ± 0.005
31.	0.97 ± 0.03	0.95 ± 0.03	0.81 ± 0.03	0.80 ± 0.02	0.79 ± 0.06	0.74 ± 0.02	0.50 ± 0.01	0.26 ± 0.03	0.04 ± 0.007
32.	0.80 ± 0.10	0.98 ± 0.00	0.77 ± 0.06	0.84 ± 0.05	0.76 ± 0.11	0.80 ± 0.04	0.61 ± 0.09	0.22 ± 0.02	0.04 ± 0.003
33.	0.89 ± 0.11	0.93 ± 0.05	0.64 ± 0.05	0.88 ± 0.06	0.87 ± 0.08	0.66 ± 0.06	0.67 ± 0.04	0.28 ± 0.02	0.02 ± 0.014
34.	0.98 ± 0.02	0.96 ± 0.04	0.98 ± 0.02	0.90 ± 0.06	0.92 ± 0.04	0.84 ± 0.08	0.65 ± 0.02	0.31 ± 0.04	0.03 ± 0.016
35.	0.91 ± 0.01	0.65 ± 0.06	0.85 ± 0.01	0.89 ± 0.03	0.83 ± 0.01	0.75 ± 0.01	0.55 ± 0.03	0.34 ± 0.01	0.09 ± 0.006
36.	1.00 ± 0.00	0.96 ± 0.04	1.00 ± 0.00	0.97 ± 0.03	0.97 ± 0.03	0.87 ± 0.11	0.76 ± 0.03	0.44 ± 0.08	0.10 ± 0.026
H37.	0.84 ± 0.04	0.55 ± 0.08	0.59 ± 0.05	0.84 ± 0.01	0.75 ± 0.01	0.75 ± 0.14	0.63 ± 0.03	0.27 ± 0.04	0.03 ± 0.000
H38.	0.81 ± 0.04	0.79 ± 0.04	0.81 ± 0.01	0.72 ± 0.05	0.65 ± 0.02	0.67 ± 0.05	0.54 ± 0.07	0.32 ± 0.02	0.06 ± 0.015
39.	0.67 ± 0.01	0.79 ± 0.08	0.72 ± 0.06	0.92 ± 0.08	0.73 ± 0.08	0.69 ± 0.07	0.40 ± 0.07	0.13 ± 0.02	0.05 ± 0.014
40.	0.74 ± 0.15	0.70 ± 0.12	0.95 ± 0.05	0.77 ± 0.12	0.67 ± 0.09	0.61 ± 0.15	0.39 ± 0.08	0.21 ± 0.00	0.05 ± 0.000

**Table 2:** Values of parameters and 95% confidence limits obtained with the IR and LQ models for 6 HRS-positive patients. The presence of the HRS response is supported by values of α_s_ higher than α_r_, the confidence limits of which do not overlap, and values of dc significantly > 0 [19].

Patient No.			IR Fit			LQ Fit	
	α_r_	α_s_	α_s_/α_r_	d_c_	β	α	β
**6**	0.64	4.58	7.2	0.17	0.009	0.62	0.014
	(0.48–0.79)	(0.91–8.24)		(0.05–0.30)	(−0.035–0.052)	(0.33–0.91)	(−0.066–0.093)
**7**	0.36	1.42	3.9	0.52	0.099	0.43	0.081
	(0.09–0.62)	(0.67–2.18)		(0.15–0.83)	(0.031–0.166)	(0.29–0.57)	(0.044–0.119)
**19**	0.62	2.53	4.1	0.38	0.039	0.62	0.040
	(0.38–0.87)	(1.17–3.90)		(0.05–0.71)	(−0.028–0.106)	(0.34–0.90)	(−0.039–0.119)
**29**	0.47	1.30	2.8	0.26	0.050	0.47	0.048
	(0.40–0.53)	(0.60–2.01)		(0.04–0.47)	(0.033–0.067)	(0.41–0.54)	(0.030–0.066)
**37**	0.42	7.71	18.4	0.13	0.106	0.43	0.104
	(0.24–0.61)	(1.11–14.31)		(0.05–0.21)	(0.05–0.156)	(0.32–0.54)	(0.074–0.134)
**38**	0.42	2.19	5.2	0.36	0.074	0.43	0.073
	(0.18–0.67)	(0.74–3.64)		(0.010–0.71)	(0.010–0.139)	(0.107–0.75)	(−0.014–0.160)

**Table 3:** Cell survival (SF) after irradiation with a single dose of 2 Gy and LDFR 4x0.5 Gy alone and in combination with carboplatin (CPL) and paclitaxel (PTX) for fibroblasts in 40 patients with head and neck cancer. Each value is mean ± SEM. Patients with numbers preceded by “H” show low-dose hyper-radiosensitivity.

Patients No.	2 Gy	4x0.5 Gy	CPL	CPL + 2 Gy	CPL + 4x0.5 Gy	PTX	PTX + 2 Gy	PTX + 4x0.5 Gy
1.	0.46 ± 0.01	0.46 ± 0.02	0.52 ± 0.01	0.16 ± 0.01	0.11 ± 0.01	0.59 ± 0.03	0.15 ± 0.02	0.13 ± 0.01
2.	0.32 ± 0.03	0.28 ± 0.02	0.35 ± 0.01	0.06 ± 0.01	0.03 ± 0.00	0.80 ± 0.06	0.21 ± 0.04	0.14 ± 0.01
3.	0.23 ± 0.04	0.18 ± 0.03	0.40 ± 0.02	0.06 ± 0.01	0.08 ± 0.01	0.64 ± 0.04	0.13 ± 0.02	0.13 ± 0.01
4.	0.35 ± 0.03	0.30 ± 0.01	0.39 ± 0.01	0.08 ± 0.01	0.06 ± 0.01	0.63 ± 0.04	0.19 ± 0.02	0.12 ± 0.02
5.	0.37 ± 0.02	0.33 ± 0.01	0.50 ± 0.04	0.13 ± 0.01	0.12 ± 0.01	0.64 ± 0.09	0.18 ± 0.04	0.20 ± 0.04
H6.	0.36 ± 0.02	0.30 ± 0.03	0.47 ± 0.04	0.19 ± 0.02	0.14 ± 0.03	0.43 ± 0.12	0.10 ± 0.04	0.10 ± 0.03
H7.	0.22 ± 0.01	0.17 ± 0.01	0.79 ± 0.03	0.12 ± 0.01	0.04 ± 0.01	0.90 ± 0.05	0.17 ± 0.01	0.17 ± 0.02
8.	0.27 ± 0.02	0.24 ± 0.01	0.46 ± 0.01	0.14 ± 0.01	0.11 ± 0.01	0.32 ± 0.03	0.04 ± 0.00	0.03 ± 0.00
9	0.19 ± 0.03	0.13 ± 0.02	0.25 ± 0.02	0.06 ± 0.01	0.05 ± 0.02	0.36 ± 0.04	0.08 ± 0.00	0.08 ± 0.01
10	0.34 ± 0.02	0.32 ± 0.02	0.47 ± 0.02	0.24 ± 0.01	0.24 ± 0.02	0.57 ± 0.02	0.27 ± 0.01	0.30 ± 0.01
11	0.33 ± 0.02	0.32 ± 0.01	0.48 ± 0.01	0.15 ± 0.01	0.12 ± 0.01	0.59 ± 0.02	0.17 ± 0.01	0.13 ± 0.01
12.	0.28 ± 0.02	0.26 ± 0.01	0.37 ± 0.00	0.03 ± 0.00	0.03 ± 0.00	0.42 ± 0.04	0.04 ± 0.00	0.03 ± 0.00
13.	0.36 ± 0.01	0.30 ± 0.00	0.30 ± 0.01	0.12 ± 0.01	0.09 ± 0.02	0.17 ± 0.01	0.07 ± 0.00	0.06 ± 0.00
14.	0.25 ± 0.03	0.24 ± 0.02	0.40 ± 0.02	0.12 ± 0.01	0.08 ± 0.01	0.42 ± 0.01	0.08 ± 0.01	0.08 ± 0.00
15.	0.32 ± 0.01	0.38 ± 0.02	0.42 ± 0.02	0.06 ± 0.01	0.06 ± 0.01	0.60 ± 0.02	0.11 ± 0.01	0.09 ± 0.00
16.	0.29 ± 0.02	0.31 ± 0.02	0.32 ± 0.01	0.10 ± 0.03	0.12 ± 0.02	0.31 ± 0.04	0.10 ± 0.01	0.11 ± 0.01
17.	0.28 ± 0.04	0.26 ± 0.02	0.45 ± 0.05	0.12 ± 0.01	0.10 ± 0.01	0.27 ± 0.02	0.06 ± 0.01	0.05 ± 0.01
18.	0.44 ± 0.01	0.49 ± 0.02	0.22 ± 0.01	0.10 ± 0.01	0.12 ± 0.01	0.62 ± 0.05	0.23 ± 0.02	0.21 ± 0.01
H19.	0.38 ± 0.05	0.45 ± 0.04	0.28 ± 0.02	0.17 ± 0.02	0.17 ± 0.00	0.61 ± 0.01	0.26 ± 0.01	0.31 ± 0.02
20.	0.49 ± 0.03	0.46 ± 0.04	0.36 ± 0.02	0.08 ± 0.01	0.10 ± 0.01	0.42 ± 0.03	0.13 ± 0.00	0.10 ± 0.01
21.	0.49 ± 0.01	0.44 ± 0.02	0.51 ± 0.02	0.16 ± 0.01	0.15 ± 0.01	0.47 ± 0.00	0.11 ± 0.00	0.12 ± 0.01
22.	0.40 ± 0.03	0.42 ± 0.06	0.40 ± 0.01	0.15 ± 0.01	0.13 ± 0.01	0.33 ± 0.02	0.11 ± 0.01	0.11 ± 0.01
23.	0.36 ± 0.02	0.35 ± 0.01	0.40 ± 0.04	0.09 ± 0.01	0.08 ± 0.01	0.48 ± 0.03	0.09 ± 0.01	0.10 ± 0.00
24.	0.29 ± 0.07	0.36 ± 0.05	0.31 ± 0.06	0.12 ± 0.02	0.11 ± 0.01	0.44 ± 0.08	0.13 ± 0.01	0.18 ± 0.02
25.	0.34 ± 0.02	0.40 ± 0.01	0.44 ± 0.02	0.14 ± 0.02	0.15 ± 0.00	0.26 ± 0.00	0.07 ± 0.00	0.08 ± 0.01
26.	0.30 ± 0.02	0.34 ± 0.03	0.37 ± 0.00	0.07 ± 0.01	0.10 ± 0.01	0.50 ± 0.01	0.11 ± 0.00	0.14 ± 0.01
27.	0.40 ± 0.01	0.45 ± 0.03	0.45 ± 0.05	0.16 ± 0.00	0.16 ± 0.01	0.58 ± 0.01	0.18 ± 0.00	0.21 ± 0.01
28.	0.37 ± 0.05	0.38 ± 0.02	0.15 ± 0.01	0.07 ± 0.00	0.09 ± 0.01	0.34 ± 0.04	0.14 ± 0.03	0.23 ± 0.02
H29.	0.35 ± 0.06	0.40 ± 0.02	0.32 ± 0.03	0.11 ± 0.01	0.13 ± 0.01	0.30 ± 0.02	0.15 ± 0.03	0.17 ± 0.01
30.	0.32 ± 0.03	0.31 ± 0.05	0.45 ± 0.05	0.12 ± 0.01	0.12 ± 0.01	0.23 ± 0.03	0.05 ± 0.01	0.05 ± 0.00
31.	0.28 ± 0.04	0.25 ± 0.02	0.59 ± 0.01	0.11 ± 0.00	0.12 ± 0.01	0.59 ± 0.04	0.14 ± 0.02	0.16 ± 0.01
32.	0.45 ± 0.05	0.46 ± 0.10	0.64 ± 0.07	0.16 ± 0.03	0.17 ± 0.02	0.32 ± 0.00	0.08 ± 0.01	0.08 ± 0.02
33.	0.29 ± 0.05	0.36 ± 0.03	0.58 ± 0.06	0.21 ± 0.01	0.24 ± 0.02	0.28 ± 0.03	0.08 ± 0.01	0.10 ± 0.01
34.	0.31 ± 0.04	0.34 ± 0.01	-	-	-	-	-	-
35.	0.36 ± 0.04	0.35 ± 0.02	0.51 ± 0.05	0.11 ± 0.02	0.09 ± 0.01	0.44 ± 0.05	0.07 ± 0.01	0.08 ± 0.03
36.	0.35 ± 0.02	0.49 ± 0.07	0.37 ± 0.00	0.20 ± 0.03	0.20 ± 0.03	0.25 ± 0.02	0.13 ± 0.02	0.19 ± 0.01
H37.	0.41 ± 0.02	0.44 ± 0.04	0.56 ± 0.03	0.22 ± 0.02	0.22 ± 0.01	0.84 ± 0.03	0.33 ± 0.01	0.33 ± 0.02
H38.	0.25 ± 0.03	0.25 ± 0.05	0.34 ± 0.04	0.20 ± 0.04	0.21 ± 0.02	0.44 ± 0.00	0.11 ± 0.00	0.20 ± 0.01
39.	0.42 ± 0.02	0.49 ± 0.04	0.43 ± 0.01	0.26 ± 0.04	0.30 ± 0.02	0.56 ± 0.05	0.34 ± 0.03	0.33 ± 0.05
40.	0.16 ± 0.02	0.15 ± 0.00	-	-	-	-	-	-

## Data Availability

All data generated and analyzed during this study are included in this article.

## Supplementary Materials

The following supporting information can be downloaded at: https://www.mdpi.com/article/10.3390/ijms27062525/s1.

## References

[1] Gleason J.F., Kudrimoti M., Van Meter E.M., Miohuddin M., Regine W.F., Valentino J., Kenady D., Arnold S.M. (2013). Low-dose fractionated radiation with induction chemotherapy for locally advanced head and neck cancer: 5-year results of a prospective phase II trial. J. Radiat. Oncol..

[2] Arnold S.M., Kudrimoti M., Dressler E.V., Gleason J.F., Silver N.L., Regine W.F., Valentino J. (2016). Using low-dose radiation potentiate the effect of induction chemotherapy in head and neck cancer: Results of a prospective phase 2 trial. Adv. Radiat. Oncol..

[3] Al-Rajhi N.M., Khalil E.M., Ahmad S., Soudy H., AlGhazi M., Fatani D.M., Memon M., Abouzied M., Khafaga Y.M. (2020). Low-dose fractionated radiation with induction docetaxel and cisplatin followed by concurrent cisplatin and radiation therapy in locally advanced nasopharyngeal cancer: A randomized phase II-III trial. Hematol. Oncol. Stem Cell Ther..

[4] Feng M., Tang Y., Fan M., Li L., Wang S., Yin Q., Ai H., Zhao S., Yin Y., Liu D. (2023). Low-dose fractionated radiotherapy combined with neoadjuvant chemotherapy for T3-4 nasopharyngeal carcinoma patients: The preliminary results of a phase II randomized controlled trial. Int. J. Radiat. Oncol. Biol. Phys..

[5] Kacorzyk U., Kentnowski M., Drosik-Rutowicz K., Słonina D., Winiarska G., Gadek A., Fidyk W., Mrocheem-Kwarciak J., Amrogowicz N., Wygoda A. (2024). Induction radiochemotherapy with low dose fractionation XRT in patients with advanced HNSCC. Radiother. Oncol..

[6] Scirocco E., Cellini F., Zamagni A., Macchia G., Deodato F., Cilla S., Strigari L., Buwenge M., Rizzo S., Cammelli S. (2021). Clinical studies on ultrafractionated chemoradiation: A systemic review. Front. Oncol..

[7] Marples B., Collis S.J. (2008). Low-dose hyper-radiosensitivity: Past, present, and future. Int. J. Radiat. Oncol. Biol. Phys..

[8] Prasanna A., Ahmed M.M., Miohiudinn M., Colema C.N. (2014). Exploiting sensitization windows of opportunity in hyper and hypo-fractionated radiation therapy. J. Thorac. Dis..

[9] Dey S., Spring P.M., Arnold S., Valentino J., Chendil D., Regine W.F., Miohiuddin M., Ahmed M.M. (2003). Low-dose fractionated radiation potentiates the effects of paclitaxel in wild-type and mutant p53 head and neck tumor cell lines. Clin. Cancer Res..

[10] Gupta S., Koru-Sengul T., Arnold S.M., Devi G.R., Mohiuddin M., Ahmed M.M. (2011). Low-dose fractionated radiation potentiates the effects of cisplatin independent of the hyper-radiation sensitivity in human lung cancer cells. Mol. Cancer Ther..

[11] Spring P.M., Arnold S.M., Shajahan S., Brown B., Dey S., Lele S.M., Valentino J., Jones R., Mohiuddin M., Ahmed M.M. (2004). Low-dose fractionated radiation potentiates the effects of taxotere in nude mice xenografts of squamous cell carcinoma of head and neck. Cell Cycle.

[12] Słonina D., Kabat D., Biesaga B., Janecka-Widła A., Szatkowski W. (2021). Chemopotentiating effects of low-dose fractionated radiation on cisplatin and paclitaxel in cervix cancer cell lines. DNA Repair..

[13] Bodgi L., Foray N. (2016). The nucleo-shuttling of the ATM protein as a basis for a novel theory of radiation response: Resolution of the linear-quadratic model. Int. J. Radiat. Biol..

[14] Berthel E., Foray N., Ferlazzo M.L. (2019). The nucleoshuttling of the ATM protein: Unified model to describe the individual response to high- and low-dose of radiation?. Cancers.

[15] Granzotto A., Benadjaoud M.A., Vogin G., Devic C., Ferlazzo M.L., Bodgi L., Pereira S., Sonzogni L., Forcheron F., Viau M. (2016). Influence of nucleoshuttling of the ATM protein in the healthy tissues’ response to radiation therapy: Toward a molecular classification of human radiosensitivity. Int. J. Radiat. Oncol. Biol. Phys..

[16] Vogin G., Bastogne T., Bodgi L., Gillet-Daubin J., Canet A., Pereira S., Foray N. (2018). The phosphorylated ATM immunofluorescence assay: A high-performance radiosensitivity assay to predict postradiation therapy overreactions. Int. J. Radiat. Oncol. Biol. Phys..

[17] Marples B., Joiner M.C. (1993). The response of Chinese hamster V79 cells to low radiation doses: Evidence of enhanced sensitivity of the whole cell population. Radiat. Res..

[18] Joiner M.C., Johns H. (1988). Renal damage in the mouse: The response to very small doses per fraction. Radiat. Res..

[19] Short S.C., Mitchell S.A., Boulton P., Woodcock M., Joiner M.C. (1999). The response of human glioma cell lines to low-dose radiation exposure. Int. J. Radiat. Biol..

[20] Słonina D., Biesaga B., Janecka A., Kabat D., Bukowska-Strakova K., Gasińska A. (2014). Low-dose hyper-radiosensitivity is not a common effect in normal asynchronous and G2-phase fibroblasts of cancer patients. Int. J. Radiat. Oncol. Biol. Phys..

[21] Martin L.M., Marples B., Lynch T.H., Hollywood D., Marignol L. (2014). Exposure to low dose ionizing radiation: Molecular and clinical consequences. Cancer Lett..

[22] Wouters B.G., Sy A.M., Skarsgard L.D. (1996). Low-dose hypersensitivity and increased radioresistance in a panel of human tumor cell lines with different radiosensitivity. Radiat. Res..

[23] Joiner M.C., Marples B., Lambin P., Short S.C., Turesson I. (2001). Low-dose hypersensitivity: Current status and possible mechanisms. Int. J. Radiat. Oncol. Biol. Phys..

[24] Słonina D., Biesaga B., Urbański K., Kojs Z. (2007). The response of primary keratinocytes and fibroblasts from cancer patients to multiple low-dose irradiations. Radiat. Res..

[25] Short S.C., Kelly J., Mayes C.R., Woodcock M., Joiner M.C. (2001). Low-dose hypersensitivity after fractionated low-dose irradiation on vitro. Int. J. Radiat. Biol..

[26] Baatout S. (2023). Radiobiology Textbook.

[27] Rothkamm K., Löbrich M. (2003). Evidence for lack of DNA double-strand break repair in human cell exposed to very low x-ray doses. Proc. Natl. Acad. Sci. USA.

[28] Suzuki K., Okada H., Yamauchi, Oka Y., Kodama S., Watanabe M. (2006). Qualitative and quantitative analysis of phosphorylated ATM foci induced by low-dose ionizing radiation. Radiat. Res..

[29] Słonina D., Kowalczyk A., Janecka-Widła A., Kabat D., Szatkowski W., Biesaga B. (2018). Low-dose hypersensitive response for residual pATM and γH2AX foci in normal fibroblasts of cancer patients. Int. J. Radiat. Oncol. Biol. Phys..

[30] Le Reun E., Bodgi L., Granzotto A., Sonzogni L., Ferlazzo M.L., Al-Choboq J., El-Nachef L., Restier-Verlet J., Berthel E., Devic C. (2022). Quantitative correlations between radiosensitivity biomarkers show that the ATM protein kinase is strongly involved in the radiotoxicities observed after radiotherapy. Int. J. Mol. Sci..

[31] Colin C., Granzotto A., Devic C., Massart C. (2011). MRE11 and H2AX biomarkers in the response to low-dose exposure: Balance between individual susceptibility to radiosensitivity and to genomic instability. Int. J. Low. Radiat..

[32] Simonsson M., Qvarnström F., Nyman J., Johansson K.A., Garmo H., Turesson I. (2008). Low-dose hypersensitive γH2AX response and infrequent apoptosis in epidermis from radiotherapy patients. Radiother. Oncol..

[33] Nuijens A.C., Oei A.L., van Oorschot B., Visser J., van Os R.M., Moerland P.D., Franken N.A.P., Rasch C.R.N., Stalpers L.J.A. (2002). Gamma-H2AX foci decay ratio as a stronger predictive factor of late radiation toxicity than dose-volume parameters in a prospective cohort of prostate cancer patients. Int. J. Radiat. Oncol. Biol. Phys..

[34] Słonina D., Biesaga B., Urbański K., Kojs Z. (2007). Low-dose radiation response of primary keratinocytes and fibroblasts from patients with cervix cancer. Radiat. Res..

