# Supplementary material for: Radiobiological Effects of Low-Dose Radiation in Normal Fibroblasts of Patients with Head and Neck Cancer Treated with Induction Chemotherapy Combined with Low-Dose Fractionated Radiation
Source: Int J Mol Sci. 2026 Mar 10;27(6):2525. doi: 10.3390/ijms27062525 (PMC13027110; doi:10.3390/ijms27062525)
Supplement: Supplementary file 1 [file ijms-27-02525-s001.zip › ijms-4167211-supplementary.pptx]

## Slide 1
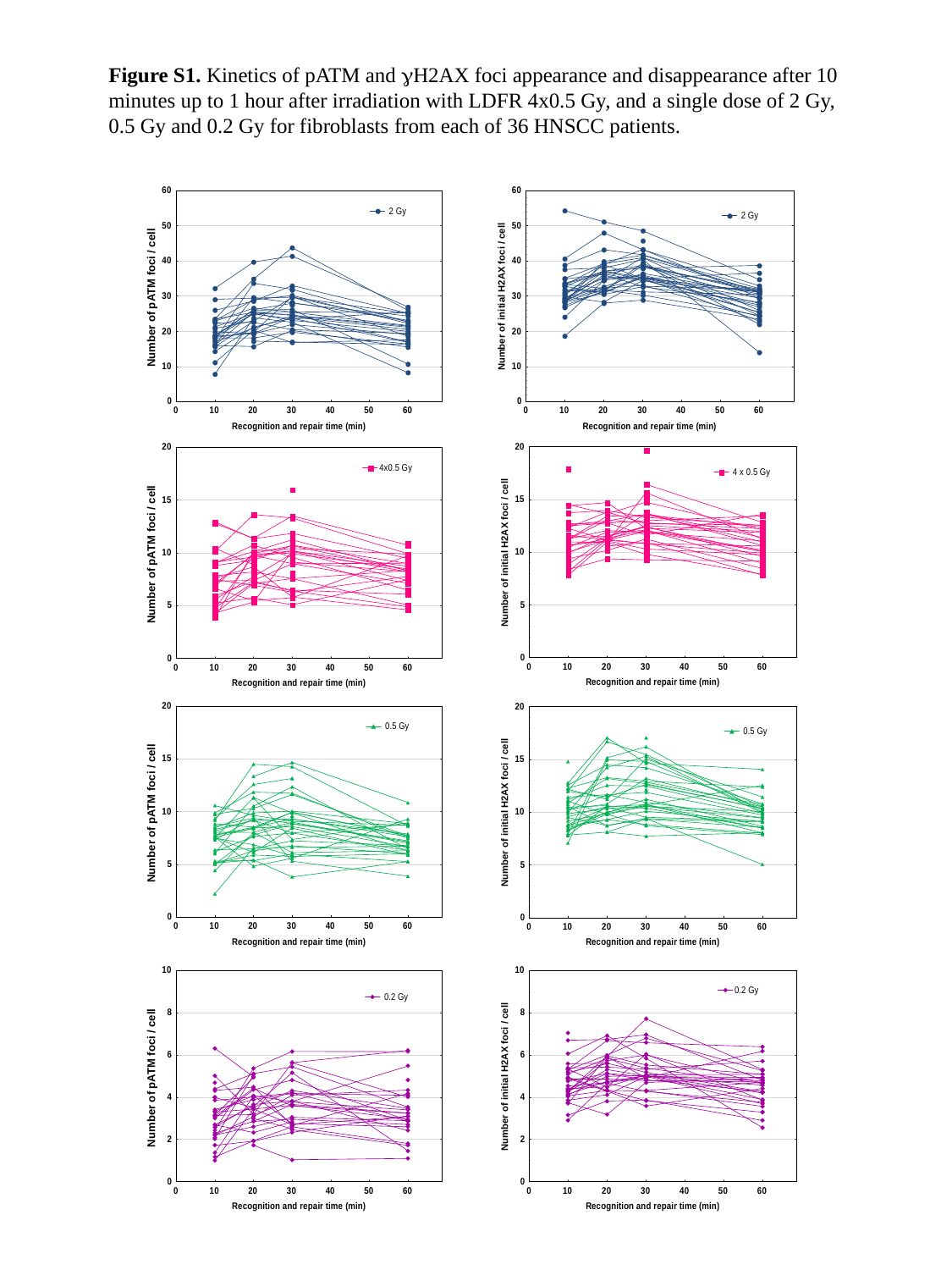

Figure S1. Kinetics of pATM and γH2AX foci appearance and disappearance after 10 minutes up to 1 hour after irradiation with LDFR 4x0.5 Gy, and a single dose of 2 Gy, 0.5 Gy and 0.2 Gy for fibroblasts from each of 36 HNSCC patients.

## Slide 2
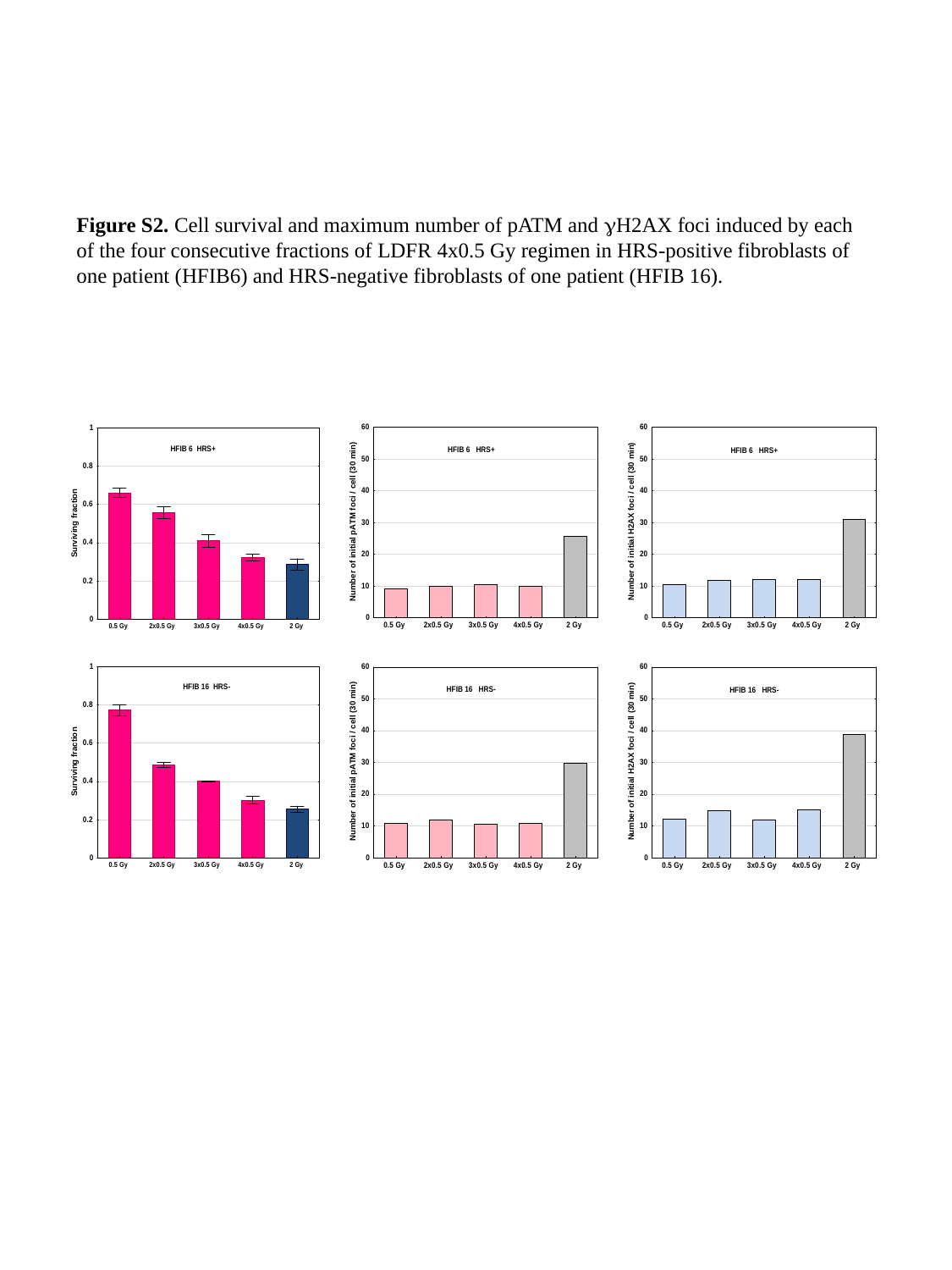

Figure S2. Cell survival and maximum number of pATM and γH2AX foci induced by each of the four consecutive fractions of LDFR 4x0.5 Gy regimen in HRS-positive fibroblasts of one patient (HFIB6) and HRS-negative fibroblasts of one patient (HFIB 16).
